# Intradermal melanocytic nevus of the external auditory canal

**DOI:** 10.1016/S1808-8694(15)31295-7

**Published:** 2015-10-20

**Authors:** Renato V. Alves, Fabiano H. Brandão, José E.P. Aquino, Maria R. M.S. Carvalho, Suzana M. Giancoli, Eduado A.P. Younes

**Affiliations:** 1 Resident Physician, Discipline of Otorhinolaryngology, Universidade de Santo Amaro; 2 Faculty Professor, Discipline of Otorhinolaryngology, Universidade de Santo Amaro; 3 Master studies in Otorhinolaryngology under course, Medical School, Santa Casa de Sao Paulo

**Keywords:** intradermal nevus, external auditory canal

## Abstract

Intradermal nevi are common benign pigmented skin tumors. Their occurrence within the external auditory canal is uncommon. The clinical and pathologic features of an intradermal nevus arising within the external auditory canal are presented, and the literature reviewed.

## INTRODUCTION

Intradermal melanocytic nevi are very common pigmented skin tumors, but they have rare occurrence inside the external auditory canal (EAC). There are few cases described in the literature.

The purpose of the present study was to report a case of intradermal melanocytic nevus of external auditory canal, discuss clinical and histopathological findings and conduct literature review.

## MATERIAL AND METHOD

**Photo 1:**
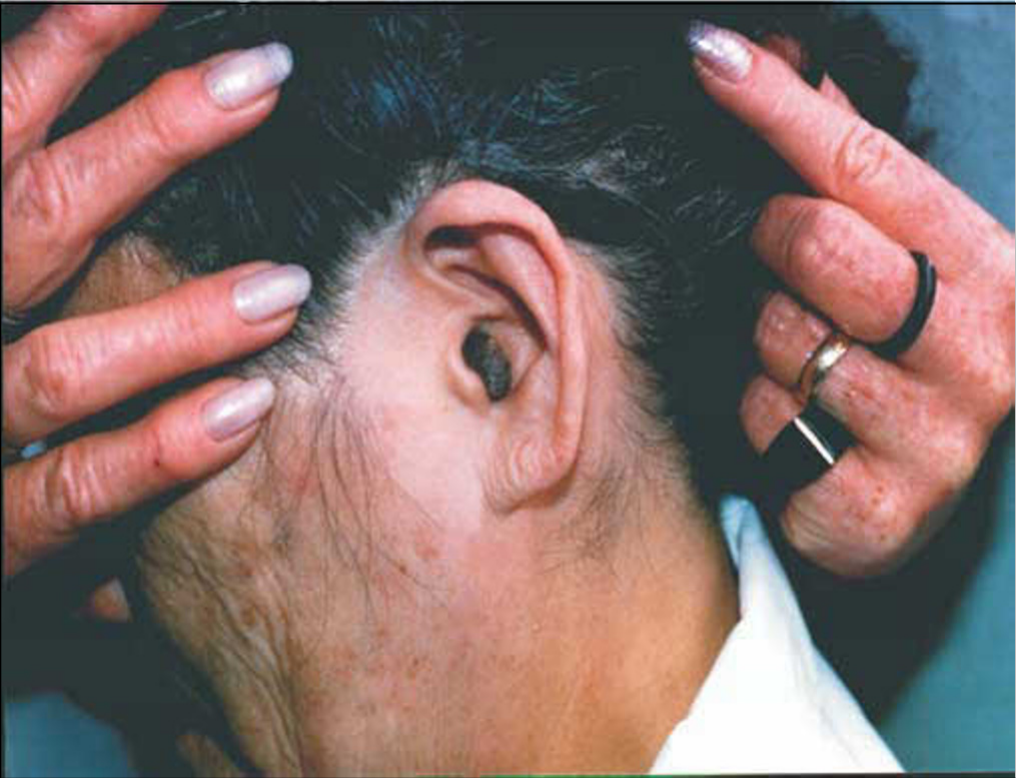
Intradermal melanocytic nevus.

SRC, 73 years old, Caucasian, Brazilian, presented left ear tumoration for approximately 20 days (Photo 1).

Otoscopy showed dark colored mass occupying the medium third extending up to the opening of the external auditory canal (EAC). It was a completely asymptomatic mass, without otorrhea, pain or hearing loss, with softened aspect, which could be easily seen in the EAC. The lesion was completely removed under local anesthesia and the surgical specimen was sent for clinical pathology. The skin on the base was cauterized to control bleeding and the remaining portion of skin from EAC was normal. Tympanic membrane was intact and absolutely normal. Surgical site was completely tumor-free 30 days after the surgery.

**Photo 2:**
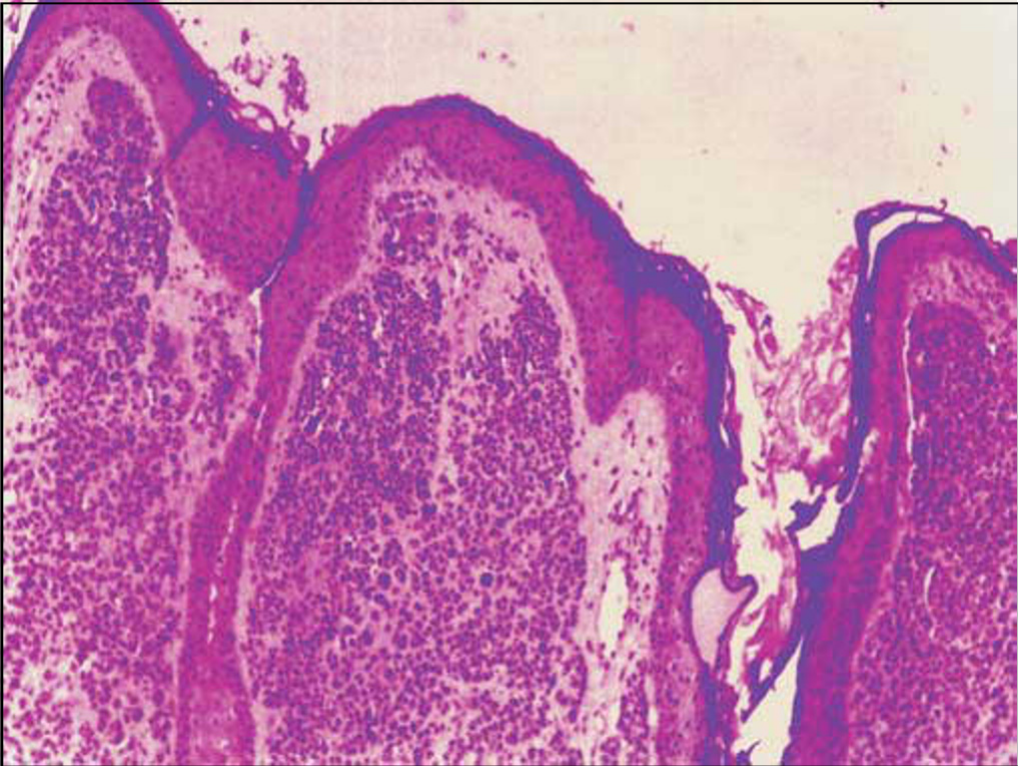
Histology section of melanocytic nevus - HE – 100 x.

***Clinical pathology analysis*** (Photo 2): Histology sections showed skin fragments with dermis that presented nevus cell on alveolar pattern, abundant cytoplasm, melanocytic pigment inside, in which nuclei were regular. There was no junctional activity.

***Diagnosis: Intradermal melanocytic nevus.***

## DISCUSSION

Nevi are pigmented benign tumors formed by proliferation of melanocytes from dermoepidermal junction. Proliferated cells form nests and migrate to dermis where they can get together to elements derived from Schwann cells. They are considered developmental defects type hamartomas. Melanocytic nevi present different clinical expressions, normally sharing pigmentation and presence of nevus cells. They are classified into three types^1^:

1. Junctional: when we can observe nests or groups of nevus cells on the basal layer, forming bulging on dermoepidermal junction.
2. Intradermal: cells are located on the dermis and are separated from the basal layer.
3. Compound: show both junctional nevus cells and other dispersed or grouped cells on the dermis.

Clinically, five macroscopic types of melanocytic nevi may be recognized^2^:

1. Flat lesions: flat pigmented spots = junctional nevus
2. Slightly elevated lesions: slight sensation of elevation over the skin = compound
3. Halo: elevated lesions with pigmented macular ring around it = compound
4. Verrucous lesions: pigmented lesions covered by fine excrescence = intradermal (some are junctional nevi)
5. Dome-like lesion: smooth, forming globous type elevation = intradermal

As to histopathology^3^, in childhood, 90% of the nevi show melanocytic proliferation on the dermoepidermal junction forming nests of nevus cells (junctional nevi). Later, nevus cells migrate to the dermis to form nests and cell columns (compound nevi). Nevus cells may extend deeply around more superficial neurovascular appendices and structures, maintaining the initial characteristics such as melanin production. The deepest ones are smaller, do not have melanin and have spindle format, taking on a neuroid aspect. Upon interrupting junctional activity, the nevus becomes intradermal. The occurrence of melanocytic nevus on the external acoustic canal skin was reported by Friedmann^4^, but most information about the topic came basically from the Japanese literature^5^.

In the EAC, intradermal nevi may present aural obstruction and conductive hearing loss, leading to accumulation of water in the external acoustic canal, acute external otitis episodes or simply found as routine otoscopic finding, as in our case.

When the nevus is symptomatic, it may be removed with excision biopsy and local anesthesia, via transcanal approach. The differential diagnosis^5^ should include inflammatory polyp, encephaloceles, foreign body granuloma, and a variety of benign and malignant neoplasms of external acoustic canal.

## CONCLUSION

Intradermal melanocytic nevi of the EAC are considerably rare. Despite their rare occurrence, the Japanese literature has some information about it. They should be excised and the material submitted to histopathological analysis.

## References

[1] Cucê LC, Festa Neto C. (1990). Manual de Dermatologia. Rio de Janeiro.

[2] Glyne LR. (1961). Tratamento dos nevos pigmentares. 4º fascículo, Relatórios Científicos Merck (Dermatologia)..

[3] Young SR, Michael H, Peter K. (1988). Intradermal nevus of the ear canal.. The Journal of Otolaryngol.

[4] Friedmann J. (1974). Pathology of the ear.

[5] Deguinec, Pulec JL. (1998). Benign nevus of the external auditory canal.. Ear Nose and Throat Journal.

